# B cell helper T cells and type 1 diabetes

**DOI:** 10.1111/sji.12943

**Published:** 2020-09-30

**Authors:** Céline Vandamme, Tuure Kinnunen

**Affiliations:** ^1^ Department of Clinical Microbiology Institute of Clinical Medicine University of Eastern Finland Kuopio Finland; ^2^ Eastern Finland Laboratory Centre (ISLAB) Kuopio Finland

**Keywords:** autoimmunity, B cells, CD4+ T cells, follicular T helper cells, peripheral T helper cells, Tfh cells, Tph cells, Type 1 diabetes

## Abstract

Type 1 diabetes is an autoimmune disease typically starting in childhood that culminates in the destruction of insulin‐producing beta cells in the pancreas. Although type 1 diabetes is considered to be a primarily T cell–mediated disease, B cells clearly participate in the autoimmune process, as autoantibodies recognizing pancreatic islet antigen commonly appear in circulation before the onset of the disease. T cells providing helper functions to B cells have recently been shown to be involved in the pathogenesis of a wide range of antibody‐associated immune disorders. These T cells include CXCR5‐positive follicular T helper (Tfh) cells, and a recently described closely related CXCR5‐negative subset coined peripheral T helper (Tph) cells. Here, we review the current state of knowledge on different B cell helper T cell subsets, focusing on their potential involvement in the development of type 1 diabetes.

## 1 INTRODUCTION

Type 1 diabetes (T1D) is a disease thought to result from an autoimmune attack damaging the beta cells in the pancreas, which leads to an insufficiency in insulin production. With the exception of exogeneous insulin replacement, no treatment is currently available to prevent or cure T1D. Circulating autoantibodies recognizing pancreatic islet antigens constitute the best biomarker currently available to identify early beta‐cell immunity and to predict the progression to clinical T1D. It has long been established that the activation and differentiation of B cells into antibody‐producing plasma cells are largely dependent on the help provided by specialized subsets of CD4^+^ helper T cells. While such T cell‐B cell interactions preferentially occur in the germinal centres of secondary lymphoid organs, growing evidence indicates that productive B cell responses can also be mounted within inflamed peripheral tissues. This review summarizes our current knowledge of the phenotypes of T cells exhibiting B cell helper function in humans, their alterations during the course of T1D and their putative contribution to T1D pathogenesis.

## 2 TYPE 1 DIABETES: AN AUTOANTIBODY‐ASSOCIATED AUTOIMMUNE DISEASE

T1D represents one of the most common chronic diseases starting in childhood, especially in Scandinavian countries where the incidence rates in children aged 0 to 14 years are among the ten highest in the world (Finland: 60.9/100,000, Sweden: 39.6/100,000, Norway: 33.6/100,000, Denmark: 27.0/100,000).^1^ Importantly, the prevalence of T1D has been continuously increasing worldwide for the last 30 years. The disease develops in genetically susceptible individuals most likely under the influence of diverse environmental triggers.^2^ The development of T1D is typically divided into three stages, the first two being presymptomatic, and it culminates in complete dependency on insulin injections to regulate blood glucose homeostasis.^2^ Despite insulin treatment, overt T1D is associated with increased mortality and morbidity, including micro‐ and macrovascular complications. Not only does T1D profoundly impact the everyday life of patients but it also puts a heavy burden on healthcare systems and societies as a whole due to the high costs associated with managing the disease. There is therefore a definite need for the development of effective preventive and/or curative therapies for T1D.

During the presymptomatic stages of T1D, which can last from months to years before clinical presentation, the detection of two or more pancreatic islet autoantibodies (AAbs), such as those against glutamic acid decarboxylase (GADA), insulin (IAA), islet antigen 2 (IA‐2A) and zinc transporter 8 (ZnT8A), is the earliest indication of ongoing beta‐cell autoimmunity and the AAbs constitute the best available biomarker for predicting T1D.^2^ Individuals positive for at least two AAbs (T1D stage 1) have almost a 50% risk of developing the disease within the next 5 years and around 80% risk within the next 15 years.^3^ This risk is further increased to almost 90% within 2 years in individuals who also develop impaired glucose tolerance (T1D stage 2).^4^ Although islet AAbs have a central role in predicting T1D progression, only indirect evidence links autoreactive B cells to T1D pathogenesis: (a) autoreactive B cells appear to contribute to islet autoimmunity in the NOD mouse model of T1D^5^, ^6^; (b) a higher frequency of CD20^+^ B cells is observed in pancreatic islets of patients with T1D who are diagnosed at a young age, and therefore likely have an aggressive form of autoimmunity^7^; (c) a partial preservation of beta‐cell function has been reported after anti‐CD20 B cell–depleting rituximab treatment^8^; and (d) B cell tolerance defects have been reported in developing naïve B cells in T1D patients.^9^ Nevertheless, since antibody production by B cells is strongly dependent on the help provided by helper T cells, the presence of islet AAbs suggests an involvement of B cells and B cell helper T cells in disease pathogenesis.

## 3 HUMAN B CELL HELPER T CELL SUBSETS: PHENOTYPE, FUNCTION AND BIOLOGICAL RELEVANCE

Follicular T helper (Tfh) cells represent the dominant memory CD4^+^ helper T cell subset capable of supporting antibody production by B cells and inducing immunoglobulin class switching.^10^ First detected in 1994 in human tonsils, their phenotype and function were unravelled in the early 2000s. Tfh cells exhibit four central features: (a) high expression of the master transcription factor BCL6, responsible for the differentiation into and the maintenance of the Tfh cell phenotype^11^, ^12^; (b) expression of CXCR5, a chemokine receptor that mediates the migration of Tfh cells to B cell follicles of secondary lymphoid organs^13^, ^14^; (c) high surface expression of ICOS and PD‐1^15^, ^16^; and (d) secretion of CXCL13 and IL‐21, cytokines facilitating the recruitment of B cells into lymphoid follicles and their differentiation into antibody‐secreting plasma cells, respectively.^15^, ^16^, ^17^, ^18^

Following the discovery of Tfh cells isolated from germinal centres of secondary lymphoid organs (GC Tfh), a circulating counterpart was later identified in blood.^10^ Circulating Tfh (cTfh) cells are memory CD4^+^ T cells that express CXCR5 but low to no BCL6.^18^, ^19^, ^20^, ^21^ They secrete IL‐21 and CXCL13 and are capable of providing help to B cells in vitro. Some cTfh cells also express PD‐1 and ICOS, albeit at lower levels than GC Tfh cells,^19^, ^20^, ^21^, ^22^ and the rare CXCR5^+^PD‐1^hi^ICOS^+^ subpopulation appears to represent recently activated cTfh cells.^23^ Paired analyses of T cells isolated from blood and lymphoid organs strongly suggest that CXCR5^+^PD‐1^+^ cTfh cells, in particular the CXCR5^+^PD‐1^hi^ICOS^+^ subpopulation, are related to GC Tfh cells both transcriptionally and clonally.^23^, ^24^, ^25^ The cTfh compartment is also phenotypically and functionally heterogeneous. On the basis of CXCR3 and CCR6 marker expression, cTfh cells can be divided into several subsets: CXCR3^+^ Th1‐like, CXCR3^‐^CCR6^‐^ Th2‐like and CXCR3^‐^CCR6^+^ Th17‐like cTfh cells that secrete the associated signature cytokines IFN‐γ, IL‐4 or IL‐17, respectively.^21^ Of these, the Th2‐ and Th17‐like cTfh cells have a stronger capacity to provide B cell help and induce class switching in vitro compared to Th1‐like cTfh cells.^21^, ^22^ Th2‐ and Th17‐like cTfh cells also appear to preferentially induce IgE and IgA production by the B cells, respectively.^21^

In 2017, a new subset of IL‐21‐producing memory CD4^+^ helper T cells was shown to be strongly expanded in the synovial fluid of seropositive rheumatoid arthritis patients.^26^ This novel population, coined peripheral T helper cells (Tph), shares important similarities with Tfh cells, such as high expression of PD‐1, ICOS, IL‐21 and CXCL13. However, Tph cells lack the expression of CXCR5, which is necessary for homing of Tfh cells to germinal centres. Instead, they express high levels of chemokine receptors, such as CCR2, CCR5 and CX3CR1, that direct them towards inflamed tissues. There, they are thought to participate in the formation of ectopic lymphoid structures (ELS) that sustain local inflammatory and antibody responses.^27^ Specifically, CXCL13 expressed by Tph cells attracts CXCR5‐expressing B cells into tissues, while IL‐21 promotes their activation and differentiation into plasma cells. IL‐21 produced by Tph cells can potentially also promote local inflammatory responses through boosting Th17 immunity as well as by increasing the cytotoxicity of CD8^+^ T cells and NK cells.^28^ A circulating counterpart of Tph cells (cTph) was also found to be expanded in the peripheral blood of rheumatoid arthritis patients.^26^

The frequencies of cTfh cells have been observed to correlate with antibody production in vivo in a variety of clinical settings. For example, increased frequencies of cTfh cells are associated with the induction of broadly neutralizing high‐avidity antibodies after influenza vaccination.^23^, ^29^ Moreover, patients affected with autoantibody‐associated autoimmune disorders, such as rheumatoid arthritis,^30^, ^31^ systemic lupus erythematosus^19^, ^20^, ^32^ and myasthenia gravis,^33^ exhibit increased frequencies of cTfh cells that correlate with autoantibody titres and disease activity. Similarly, frequencies of the more recently identified cTph cells also appear to be increased in autoimmune disorders, such as rheumatoid arthritis,^26^, ^34^ systemic lupus erythematosus^35^, ^36^, ^37^ and coeliac disease.^38^

## 4 ALTERATIONS OF CIRCULATING TFH AND TPH CELLS IN T1D

Several independent studies have investigated the frequencies of cTfh cells in the context of T1D (Table 1). The clinical cohorts studied have mainly comprised Caucasians, encompassing both paediatric and adult subjects at different stages of the disease: AAb^+^ at‐risk individuals with normal glucose tolerance (T1D Stage 1), AAb^+^ at‐risk individuals with impaired glucose tolerance (T1D Stage 2) and new‐onset or long‐standing T1D patients (T1D Stage 3). In most occasions, the control group has composed of age‐matched AAb‐ healthy individuals, with some more stringently controlled studies also matching for HLA genotype.

**Table 1:** Studies addressing cTfh and cTph cells in T1D cohorts

Clinical cohorts studied	cTfh/cTph definitions used	Main findings	Reference
Asian cohort 54 recent‐onset T1D patients (<2 years from disease onset) 31 age‐ and sex‐matched controls	cTfh: CD4^+^CXCR5^+^ICOS^+^	Increased frequency of cTfh cells in patients. Elevated levels of serum IL‐21 in patients.	[39]
Caucasian cohort 24 long‐standing T1D patients 15 age‐ and sex‐matched controls	cTfh: CD4^+^CD45RA^‐^CXCR5^+^ (ICOS^+^)	Increased frequency of cTfh cells in patients. Increased expression of Tfh markers *CXCR5, ICOS, PDCD1* and *BCL6* in CD4^+^CD45RA^‐^ T cells from patients. Increased IL‐21‐production in CD4^+^ T cells from patients.	[40]
Caucasian cohort 30 long‐standing T1D patients 32 age‐ and sex‐matched controls	cTfh: CD4^+^CD45RA^‐^CXCR5^+^ PD1^+^CCR6^‐^	Increased frequency of cTfh cells in patients. Increased IL‐21 production in CD4^+^CD45RA^‐^ T cells from patients.	[41]
Caucasian paediatric and adult cohort 14 AAb^+^ subjects (5 with recent autoimmunity: AAb‐positivity for < 5 years) 9 AAb^‐^ controls	cTfh: CD4^+^CD45RA^‐^CXCR5^+^ PD^hi^CCR7^lo^	Increased frequency of cTfh cells in AAb^+^ subjects with recent autoimmunity (<5 years) compared to AAb‐ controls and AAb^+^ subjects with longer autoimmunity (>5 years) Increased frequency of insulin‐specific CD4^+^ T cells with a CD4^+^CXCR5^+^ phenotype in AAb^+^ subjects with recent autoimmunity	[44]
Caucasian paediatric cohort 54 children with newly diagnosed T1D (<7 days) 58 AAb^+^ children 15 AAb^+^ children with IGT 149 age‐ and HLA‐matched AAb‐ controls	cTfh: CD4^+^CD45RA^‐^CXCR5^+^ PD1^+^ICOS^+^	Increased frequency of cTfh cells in children with T1D and in AAb^+^ children with IGT who were positive for >2 AAbs. cTfh cell frequency peaks around the clinical manifestation of T1D.	[42]
Caucasian paediatric cohort 29 children with recent‐onset T1D (< 2 months) 29 AAb^+^ children 24 AAb^‐^ age‐matched controls	cTfh: CD4^+^CXCR5^+^(CXCR3^‐^CCR6^‐^)	Slightly elevated frequencies of CXCR3^‐^CCR6^‐^ cTfh cells in children with newly diagnosed T1D	[43]
Caucasian paediatric cohort 44 children with newly diagnosed T1D (<7 days) 40 AAb^+^ children (of which 15 progressed to clinical T1D) 84 age‐ and HLA‐matched AAb‐ controls	cTph: CD4^+^CD45RA^‐^CXCR5^‐^PD‐1^hi^	Increased frequency of cTph cells in children with newly diagnosed T1D and in AAb^+^ children who progressed to T1D.	[45]

Investigators have utilized variable markers to define cTfh cells by flow cytometry. At minimum, cTfh cells have been defined as CD4^+^CXCR5^+^, but additional markers, in particular PD‐1 and ICOS, have been used to define cTfh cells in some studies. All studies report elevated frequencies of cTfh cells in adult patients with recent‐onset or long‐standing T1D compared to healthy controls, some along with increased IL‐21 production by CD4^+^ T cells.^39^, ^40^, ^41^ Xu et al additionally showed that cTfh cell frequencies were decreased in patients with recent‐onset T1D after B cell–depleting rituximab treatment.^39^ Elevated frequencies of cTfh cells have also been reported in children with newly diagnosed T1D.^42^, ^43^ For the presymptomatic stage, Serr *et al* were first to show that cTfh cell frequencies were increased in AAb^+^ at‐risk subjects, but only in a small cohort with a shorter duration of islet autoimmunity (<5 years of AAb‐positivity).^44^ They further demonstrated an increase in insulin‐specific blood CD4^+^ T cells with a CD4^+^CXCR5^+^ Tfh phenotype in this same cohort. To our knowledge, our own study represents the largest paediatric at‐risk cohort in which cTfh cells have been assessed.^42^ We observed elevated frequencies of cTfh cells in AAb^+^ at‐risk children with impaired glucose tolerance (T1D stage 2) but not in those without (T1D stage 1). Longitudinal analyses indicated that the frequency of cTfh cells peaked around disease onset in AAb^+^ children who progressed to clinical T1D. We also reported that positivity for multiple islet AAbs at disease onset associated with increased cTfh cell frequencies. In line with our results, Vecchione *et al* did not detect any change in cTfh cell frequencies in paediatric AAb^+^ subjects compared to controls.^43^

To our knowledge, our group is the only one so far to have investigated the new cTph population in the context of T1D.^45^ In our study, we observed elevated frequencies of cTph cells in children with newly diagnosed T1D as well as in AAb^+^ at‐risk children who later progressed to T1D, but not in AAb^+^ children who did not progress.^45^

Taken together, these studies suggest that cTfh and cTph cell frequencies are elevated in individuals with T1D. At the presymptomatic stage, the results are more variable, and increased frequencies have only been reported in a subset of AAb^+^ at‐risk individuals who have either recent onset of autoimmunity or who later progressed to T1D.

## 5 ROLE OF TFH AND TPH CELLS IN T1D PATHOGENESIS: INSIGHTS FROM MOUSE MODELS AND OTHER AUTOIMMUNE DISEASES

Although elevated frequencies of cTfh and cTph cells are observed at different stages of T1D development, the involvement of Tfh and Tph cells in T1D pathogenesis is still unresolved. In particular, it is unclear whether Tfh and Tph cells simply support the production of islet autoantibodies by autoreactive B cells or whether they have a more direct role in promoting autoimmunity at the level of inflamed islets.

Some insight into these questions can be obtained from mouse models of T1D. B cell infiltration and the generation of ELS are common features of autoimmune insulitis in NOD mice.^46^, ^47^, ^48^ Moreover, IL‐21, the central cytokine produced by Tfh and Tph cells, appears to play a pivotal role in autoimmune diabetes: IL‐21R‐deficient NOD mice are protected from diabetes, whereas IL‐21 overexpression in beta cells precipitates the disease in diabetes‐resistant mice.^49^ In a transgenic T cell receptor (TCR) model, a strong Tfh signature was observed in islet antigen‐specific CD4^+^ T cells isolated from pancreatic lymph nodes and these Tfh cells induced diabetes in adoptive transfer experiments.^40^ Another study identified CXCR5^‐^ICOS^+^ IL‐21‐producing CD4^+^ T cells to be enriched in the inflamed pancreas of NOD mice.^50^ These cells exhibited high resemblance to human Tph cells, as they were able to support antibody production by B cells in vitro. In addition, they directly contributed to disease pathogenesis by enhancing the proliferation and survival of diabetogenic CD8^+^ T cells through IL‐21 secretion in vivo.

Although ELS have not been detected in pancreases of human T1D patients, variable levels of B cell and CD4^+^ and CD8^+^ T cell infiltration can be observed in inflamed islets.^7^ Intriguingly, children diagnosed with T1D at a young age harbour more B cells in their inflamed islets compared to individuals diagnosed at a later age, and this is accompanied by a more pronounced loss of insulin‐producing beta cells.^7^ This observation suggests that the presence of B cells in islets is a hallmark of more aggressive inflammatory lesions. Although the phenotype of infiltrating CD4^+^ T cells in inflamed human islets has not been resolved, comparison to other autoimmune diseases characterized by autoantibody production suggests that they may contain T cells with a Tph phenotype. In both rheumatoid arthritis and coeliac disease, CD4^+^ T cells detected in inflamed synovia or gut, respectively, display a CXCR5^‐^PD1^hi^ phenotype and produce IL‐21 and CXCL13.^26^, ^38^ Additional work has recently provided insight into the developmental origin of Tph cells detected in inflamed tissues. A paired analysis of lymph node and blood samples from HIV‐infected patients demonstrated TCR repertoire sharing and epigenetic similarities between GC Tfh, GC Tph and cTph cells.^51^ Altogether, these results support a model in which CXCR5^‐^ Tph cells derive from CXCR5^+^ Tfh cells during GC reaction and upregulate a migratory transcriptional programme that allows them to egress from lymph nodes and home to inflamed tissues.^51^ This close developmental relationship is also supported by the finding that cTfh and cTph frequencies correlate strongly with each other.^45^

**Figure 1:**
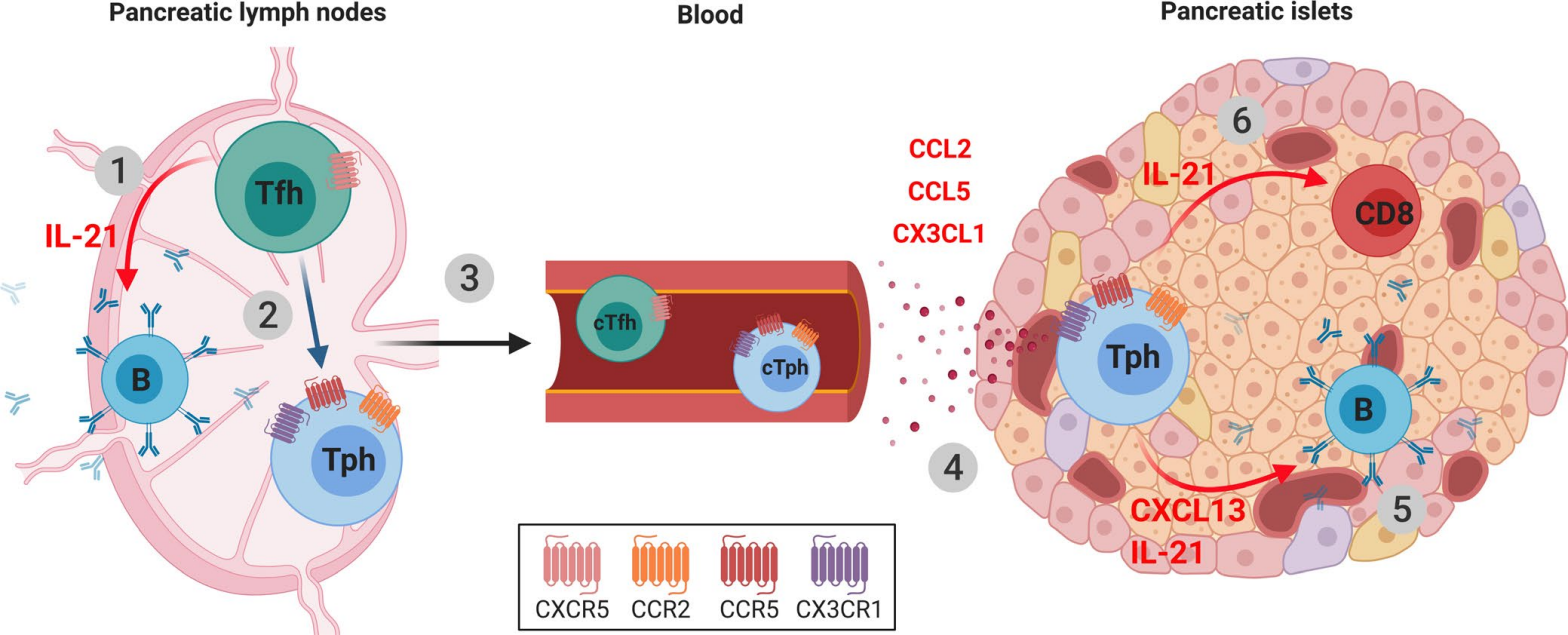
A putative model for the involvement of B cell helper T cells in T1D pathogenesis. 1) CXCR5^+^ islet antigen‐specific Tfh cells are induced in pancreatic lymph nodes and support islet AAb production by autoreactive B cells. 2) A subset of Tfh cells downregulates CXCR5 and upregulates chemokine receptors (CCR2, CCR5 and CX3CR1) to become Tph cells. 3) Following Tfh activation in GCs, cTfh and cTph become detectable in peripheral blood. 4) Tph cells home to inflamed pancreatic islets due to the presence of CCL2, CCL5 and CX3CL1. 5) In islets, Tph cells produce CXCL13, which attracts B cells, and IL‐21, which promotes B cell activation and local AAb production. 6) IL‐21 may also support the proliferation and survival of autoreactive cytotoxic CD8^+^ T cells which further drive beta‐cell destruction

Based on the available evidence, we propose the following hypothetical model for the role of Tfh/Tph cells in the pathogenesis of T1D (Figure 1). (a) Following a breach in central and peripheral tolerance, Tfh cells specific for islet antigens are induced in pancreatic lymph nodes, where they give help to autoreactive B cells to produce islet AAbs. (b) Some of the Tfh cells lose CXCR5 expression and upregulate chemokine receptors such as CCR2, CCR5 and CX3CR1 to become Tph cells that egress from lymph nodes. (c) cTfh and cTph can be detected in blood as a consequence of Tfh activation in GCs. (d) CCL2, CCL5 and CX3CL1 attract Tph cells to inflamed islets. (e) In situ, Tph cells produce CXCL13 that attracts B cells into inflamed islets and IL‐21 that promotes B cell maturation, leading to local AAb production. Activated B cells produce cytokines and can function as antigen‐presenting cells to further amplify local immune activation (f). IL‐21 produced by Tph cells may also support the proliferation and survival of cytotoxic CD8^+^ T cells, which represent the main immune cell subset infiltrating inflamed islets.^7^ Islet‐infiltrating CD8^+^ T cells display reactivity to islet autoantigens^52^ and are widely believed to be the terminal mediators of beta‐cell destruction.

## 6 CONCLUDING REMARKS AND PERSPECTIVES

While our understanding of T cell subsets providing help to B cells in health and disease has expanded beyond the original identification of GC Tfh cells, the complex interactions between T cells and B cells in secondary lymphoid organs and inflamed tissues remain to be fully resolved. In the context of T1D, a key open question is whether CD4^+^ T cells with a Tph phenotype truly exist in inflamed islets. Another important question is whether cTfh and cTph cell expansions in blood represent autoantigen‐specific cells, as recently observed in coeliac disease,^38^ or whether they represent unspecific T cells expanded through bystander activation during active autoimmunity. Novel high‐throughput technologies, such as high‐dimensional flow/mass cytometry, TCR repertoire analyses and single‐cell transcriptomic profiling, offer exciting new opportunities to answer these technically challenging questions. The potential of using cTfh/cTph cells as biomarkers of T1D progression^39^, ^40^, ^41^, ^42^, ^43^, ^44^, ^45^ must also be validated with additional longitudinal cohorts. Finally, Tfh/Tph activation pathways can also be envisioned as prospective targets for immunotherapy of T1D.^53^ The recently completed phase 2 clinical trial testing anti‐IL‐21 in patients with newly diagnosed T1D (NCT02443155) may give first insights into the feasibility of this approach.

## CONFLICT OF INTEREST

The authors have no conflict of interests to declare.

## AUTHOR CONTRIBUTIONS

Both authors contributed equally to the writing of this manuscript.

## References

[1] Patterson CC , Harjutsalo V , Rosenbauer J , et al. Trends and cyclical variation in the incidence of childhood type 1 diabetes in 26 European centres in the 25 year period 1989–2013: a multicentre prospective registration study. Diabetologia. 2019;62(3):408‐417. 30483858 10.1007/s00125-018-4763-3

[2] Ilonen J , Lempainen J , Veijola R . The heterogeneous pathogenesis of type 1 diabetes mellitus. Nat Rev Endocrinol. 2019;15(11):635‐650. 31534209 10.1038/s41574-019-0254-y

[3] Ziegler AG , Rewers M , Simell O , et al. Seroconversion to multiple islet autoantibodies and risk of progression to diabetes in children. JAMA. 2013;309(23):2473‐2479. 23780460 10.1001/jama.2013.6285 PMC4878912

[4] Helminen O , Aspholm S , Pokka T , et al. OGTT and random plasma glucose in the prediction of type 1 diabetes and time to diagnosis. Diabetologia. 2015;58(8):1787‐1796. 25985749 10.1007/s00125-015-3621-9

[5] Serreze DV , Chapman HD , Varnum DS , et al. B lymphocytes are essential for the initiation of T cell‐mediated autoimmune diabetes: analysis of a new speed congenic stock of NOD.Ig mu null mice. J Exp Med. 1996;184(5):2049‐2053. 8920894 10.1084/jem.184.5.2049 PMC2192892

[6] Hu C , Rodriguez‐Pinto D , Du W , et al. Treatment with CD20‐specific antibody prevents and reverses autoimmune diabetes in mice. J Clin Invest. 2007;117(12):3857‐3867. 18060033 10.1172/JCI32405 PMC2096456

[7] Leete P , Willcox A , Krogvold L , et al. Differential insulitic profiles determine the extent of β‐cell destruction and the age at onset of type 1 diabetes. Diabetes. 2016;65(5):1362‐1369. 26858360 10.2337/db15-1615

[8] Pescovitz MD , Greenbaum CJ , Krause‐Steinrauf H , et al. Rituximab, B‐lymphocyte depletion, and preservation of beta‐cell function. N Engl J Med. 2009;361(22):2143‐2152. 19940299 10.1056/NEJMoa0904452 PMC6410357

[9] Menard L , Saadoun D , Isnardi I , et al. The PTPN22 allele encoding an R620W variant interferes with the removal of developing autoreactive B cells in humans. J Clin Invest. 2011;121(9):3635‐3644. 21804190 10.1172/JCI45790 PMC3163953

[10] Crotty S . T Follicular helper cell biology: a decade of discovery and diseases. Immunity. 2019;50(5):1132‐1148. 31117010 10.1016/j.immuni.2019.04.011 PMC6532429

[11] Johnston RJ , Poholek AC , DiToro D , et al. Bcl6 and Blimp‐1 are reciprocal and antagonistic regulators of T follicular helper cell differentiation. Science. 2009;325(5943):1006‐1010. 19608860 10.1126/science.1175870 PMC2766560

[12] Nurieva RI , Chung Y , Martinez GJ , et al. Bcl6 mediates the development of T follicular helper cells. Science. 2009;325(5943):1001‐1005. 19628815 10.1126/science.1176676 PMC2857334

[13] Breitfeld D , Ohl L , Kremmer E , et al. Follicular B helper T cells express Cxc chemokine receptor 5, localize to B cell follicles, and support immunoglobulin production. J Exp Med. 2000;192(11):1545‐1552. 11104797 10.1084/jem.192.11.1545 PMC2193094

[14] Schaerli P , Willimann K , Lang AB , Lipp M , Loetscher P , Moser B . Cxc chemokine receptor 5 expression defines follicular homing T CElls with B cell helper function. J Exp Med. 2000;192(11):1553‐1562. 11104798 10.1084/jem.192.11.1553 PMC2193097

[15] Rasheed A‐U , Rahn H‐P , Sallusto F , Lipp M , Müller G . Follicular B helper T cell activity is confined to CXCR5hiICOShi CD4 T cells and is independent of CD57 expression. Eur J Immunol. 2006;36(7):1892‐1903. 16791882 10.1002/eji.200636136

[16] Chtanova T , Tangye SG , Newton R , et al. T follicular helper cells express a distinctive transcriptional profile, reflecting their role as non‐Th1/Th2 effector cells that provide help for B cells. J Immunol. 2004;173(1):68‐78. 15210760 10.4049/jimmunol.173.1.68

[17] Kroenke MA , Eto D , Locci M , et al. Bcl6 and Maf cooperate to instruct human follicular helper CD4 T cell (Tfh) differentiation. J Immunol. 2012;188(8):3734‐3744. 22427637 10.4049/jimmunol.1103246 PMC3324673

[18] Chevalier N , Jarrossay D , Ho E , et al. CXCR5 expressing human central memory CD4 T cells and their relevance for humoral immune responses. J Immunol. 2011;186(10):5556‐5568. 21471443 10.4049/jimmunol.1002828

[19] He J , Tsai LM , Leong YA , et al. Circulating precursor CCR7loPD‐1hi CXCR5+ CD4+ T cells indicate Tfh cell activity and promote antibody responses upon antigen reexposure. Immunity. 2013;39(4):770‐781. 24138884 10.1016/j.immuni.2013.09.007

[20] Simpson N , Gatenby PA , Wilson A , et al. Expansion of circulating T cells resembling follicular helper T cells is a fixed phenotype that identifies a subset of severe systemic lupus erythematosus. Arthritis Rheum. 2010;62(1):234‐244. 20039395 10.1002/art.25032

[21] Morita R , Schmitt N , Bentebibel S‐E , et al. Human blood CXCR5+CD4+ T cells are counterparts of T follicular cells and contain specific subsets that differentially support antibody secretion. Immunity. 2011;34(1):108‐121. 21215658 10.1016/j.immuni.2010.12.012 PMC3046815

[22] Locci M , Havenar‐Daughton C , Landais E , et al. Human circulating PD‐1+CXCR3−CXCR5+ memory Tfh cells are highly functional and correlate with broadly neutralizing HIV antibody responses. Immunity. 2013;39(4):758‐769. 24035365 10.1016/j.immuni.2013.08.031 PMC3996844

[23] Heit A , Schmitz F , Gerdts S , et al. Vaccination establishes clonal relatives of germinal center T cells in the blood of humans. J Exp Med. 2017;214(7):2139‐2152. 28637884 10.1084/jem.20161794 PMC5502430

[24] Hill DL , Pierson W , Bolland DJ , et al. The adjuvant GLA‐SE promotes human Tfh cell expansion and emergence of public TCRβ clonotypes. J Exp Med. 2019;216(8):1857‐1873. 31175140 10.1084/jem.20190301 PMC6683991

[25] Vella LA , Buggert M , Manne S , et al. T follicular helper cells in human efferent lymph retain lymphoid characteristics. J Clin Invest. 2019;129(8):3185‐3200. 31264971 10.1172/JCI125628 PMC6668682

[26] Rao DA , Gurish MF , Marshall JL , et al. Pathologically expanded peripheral T helper cell subset drives B cells in rheumatoid arthritis. Nature. 2017;542(7639):110‐114. 28150777 10.1038/nature20810 PMC5349321

[27] Rao DA . T Cells That Help B Cells in Chronically Inflamed Tissues. Front Immunol. 2018;9. 10.3389/fimmu.2018.01924 PMC6115497 30190721

[28] Spolski R , Leonard WJ . Interleukin‐21: basic biology and implications for cancer and autoimmunity. Annu Rev Immunol. 2008;26(1):57‐79. 17953510 10.1146/annurev.immunol.26.021607.090316

[29] Bentebibel S‐E , Khurana S , Schmitt N , et al. ICOS+PD‐1+CXCR3+ T follicular helper cells contribute to the generation of high‐avidity antibodies following influenza vaccination. Sci Rep. 2016;6:26494. 27231124 10.1038/srep26494 PMC4882544

[30] Arroyo‐Villa I , Bautista‐Caro M‐B , Balsa A , et al. Constitutively altered frequencies of circulating follicullar helper T cell counterparts and their subsets in rheumatoid arthritis. Arthritis Res Ther. 2014;16(6):500. 25475240 10.1186/s13075-014-0500-6 PMC4275955

[31] Liu R , Wu Q , Su D , et al. A regulatory effect of IL‐21 on T follicular helper‐like cell and B cell in rheumatoid arthritis. Arthritis Res Ther. 2012;14(6):R255. 23176102 10.1186/ar4100 PMC3674600

[32] Choi J‐Y , Ho JH , Pasoto SG , et al. Circulating follicular helper‐like T cells in systemic lupus erythematosus: association with disease activity. Arthritis Rheumatol. 2015;67(4):988‐999. 25581113 10.1002/art.39020 PMC4450082

[33] Zhang C‐J , Gong Y , Zhu W , et al. Augmentation of circulating follicular helper T cells and their impact on autoreactive B cells in myasthenia gravis. J Immunol. 2016;197(7):2610‐2617. 27543617 10.4049/jimmunol.1500725

[34] Fortea‐Gordo P , Nuño L , Villalba A , et al. Two populations of circulating PD‐1hiCD4 T cells with distinct B cell helping capacity are elevated in early rheumatoid arthritis. Rheumatology. 2019;58(9):1662‐1673. 31056653 10.1093/rheumatology/kez169

[35] Bocharnikov AV , Keegan J , Wacleche VS , et al. PD‐1^hi^CXCR5^–^ T peripheral helper cells promote B cell responses in lupus via MAF and IL‐21. JCI Insight. 2019;4(20). 10.1172/jci.insight.130062 PMC6824311 31536480

[36] Makiyama A , Chiba A , Noto D , et al. Expanded circulating peripheral helper T cells in systemic lupus erythematosus: association with disease activity and B cell differentiation. Rheumatology. 2019;58(10):1861‐1869. 30879065 10.1093/rheumatology/kez077

[37] Lin J , Yu Y , Ma J , Ren C , Chen W . PD‐1+CXCR5−CD4+T cells are correlated with the severity of systemic lupus erythematosus. Rheumatology. 2019;58(12):2188‐2192. 31180450 10.1093/rheumatology/kez228

[38] Christophersen A , Lund EG , Snir O , et al. Distinct phenotype of CD4 + T cells driving celiac disease identified in multiple autoimmune conditions. Nat Med. 2019;25(5):734. 30911136 10.1038/s41591-019-0403-9 PMC6647859

[39] Xu X , Shi Y , Cai Y , et al. Inhibition of increased circulating Tfh cell by anti‐CD20 monoclonal antibody in patients with type 1 diabetes. PLoS One. 2013;8(11):e79858. 24278195 10.1371/journal.pone.0079858 PMC3835920

[40] Kenefeck R , Wang CJ , Kapadi T , et al. Follicular helper T cell signature in type 1 diabetes. J Clin Invest. 2015;125(1):292‐303. 25485678 10.1172/JCI76238 PMC4382272

[41] Ferreira RC , Simons HZ , Thompson WS , et al. IL‐21 production by CD4+ effector T cells and frequency of circulating follicular helper T cells are increased in type 1 diabetes patients. Diabetologia. 2015;58(4):781‐790. 25652388 10.1007/s00125-015-3509-8 PMC4351433

[42] Viisanen T , Ihantola E‐L , Näntö‐Salonen K , et al. Circulating CXCR5+PD‐1+ICOS+ follicular T helper cells are increased close to the diagnosis of type 1 diabetes in children with multiple autoantibodies. Diabetes. 2017;66(2):437‐447. 28108610 10.2337/db16-0714

[43] Vecchione A , Di Fonte R , Gerosa J , et al. Reduced PD‐1 expression on circulating follicular and conventional FOXP3+ Treg cells in children with new onset type 1 diabetes and autoantibody‐positive at‐risk children. Clin Immunol. 2019;211:108319. 31794865 10.1016/j.clim.2019.108319

[44] Serr I , Fürst RW , Ott VB , et al. miRNA92a targets KLF2 and the phosphatase PTEN signaling to promote human T follicular helper precursors in T1D islet autoimmunity. Proc Natl Acad Sci USA. 2016;113(43):E6659‐E6668. 27791035 10.1073/pnas.1606646113 PMC5087025

[45] Ekman I , Ihantola E‐L , Viisanen T , et al. Circulating CXCR5−PD‐1hi peripheral T helper cells are associated with progression to type 1 diabetes. Diabetologia. 2019;62(9):1681‐1688. 31270583 10.1007/s00125-019-4936-8 PMC6677711

[46] Lee Y , Chin RK , Christiansen P , et al. Recruitment and activation of naive T cells in the islets by lymphotoxin beta receptor‐dependent tertiary lymphoid structure. Immunity. 2006;25(3):499‐509. 16934497 10.1016/j.immuni.2006.06.016

[47] Kendall PL , Yu G , Woodward EJ , Thomas JW . Tertiary lymphoid structures in the pancreas promote selection of B lymphocytes in autoimmune diabetes. J Immunol. 2007;178(9):5643‐5651. 17442947 10.4049/jimmunol.178.9.5643

[48] Penaranda C , Tang Q , Ruddle NH , Bluestone JA . Prevention of diabetes by FTY720‐mediated stabilization of Peri‐Islet tertiary lymphoid organs. Diabetes. 2010;59(6):1461‐1468. 20299465 10.2337/db09-1129 PMC2874707

[49] Sutherland APR , Van Belle T , Wurster AL , et al. Interleukin‐21 is required for the development of type 1 diabetes in NOD mice. Diabetes. 2009;58(5):1144‐1155. 19208913 10.2337/db08-0882 PMC2671036

[50] McGuire HM , Vogelzang A , Ma CS , et al. A subset of interleukin‐21+ chemokine receptor CCR9+ T helper cells target accessory organs of the digestive system in autoimmunity. Immunity. 2011;34(4):602‐615. 21511186 10.1016/j.immuni.2011.01.021

[51] Del Alcazar D , Wang Y , He C , et al. Mapping the lineage relationship between CXCR5+ and CXCR5− CD4+ T cells in HIV‐infected human lymph nodes. Cell Rep. 2019;28(12):3047‐3060.e7. 31533030 10.1016/j.celrep.2019.08.037 PMC6878759

[52] Coppieters KT , Dotta F , Amirian N , et al. Demonstration of islet‐autoreactive CD8 T cells in insulitic lesions from recent onset and long‐term type 1 diabetes patients. J Exp Med. 2012;209(1):51‐60. 22213807 10.1084/jem.20111187 PMC3260877

[53] Yan L , de Leur K , Hendriks RW , et al. T follicular helper cells as a new target for immunosuppressive therapies. Front Immunol. 2017;8. 10.3389/fimmu.2017.01510 PMC5681999 29163552

